# Music aesthetic teaching and emotional visualization under emotional teaching theory and deep learning

**DOI:** 10.3389/fpsyg.2022.911885

**Published:** 2022-07-14

**Authors:** Yang Li

**Affiliations:** School of Music, Shandong Normal University, Jinan, China

**Keywords:** emotional teaching theory, deep neural network, music aesthetic teaching, emotion visualization system, music teaching

## Abstract

The study aims to overcome the shortcomings of the traditional music teaching system, for it cannot analyze the emotions of music works and does not have the advantages in music aesthetic teaching. First, the relevant theories of emotional teaching are expounded and the important roles of emotional teaching and aesthetic teaching in shaping students’ personalities are described. Second, a music emotion classification model based on the deep neural network (DNN) is proposed, and it can accurately classify music emotions through model training. Finally, according to the emotional teaching theory and the model based on DNN, a visual system of music teaching is designed for visualizing the emotions, which is helpful to students’ understanding of music works and the improvement of teaching effect. The results show that: (1) the teaching system designed has five parts, namely the audio input layer, emotion classification layer, virtual role perception layer, emotion expression layer, and output layer. The system can classify the emotions of the current input audio and map it to the virtual characters for emotional expression. Finally, the emotions are displayed to the students through the display screen layer to realize the visualization of the emotions of music works, so that the students can intuitively feel the emotional elements in the works. (2) The accuracy of the music emotion classification model based on DNN is more than 3.4% higher than other models and has better performance. The study provides important technical support for the upgrading of the teaching system and improving the quality of music aesthetic teaching.

## Introduction

Music is usually used for relaxing and cultivating sentiment, and now it is introduced for school aesthetic teaching (Silva, 2020). Every school opens music courses, and professional teachers teach their students to identify the cultural information and emotional elements contained in excellent music works in classrooms after in-depth interpretation. And practical activities, such as learning singing and audio appreciation, are carried out so that students can deepen their understanding and finally resonate with the works (Burak, 2019). The objective of music teaching is to help students feel the rich cultural and emotional elements through appreciating classic music works, improve their aesthetics and gradually establish correct aesthetic values and a sound personality (Johnson, 2017). Since emotion is the most important element in music teaching except cultural information, exploring students’ psychology and a reasonable way to carry out emotional teaching in the learning process is the problem to be solved in music teaching.

Ye (2020) investigated the current situation of music classroom teaching in Colleges and Universities (CAUs) through Questionnaire Survey (QS). They found problems in music classroom teaching, such as lack of demonstration, evaluation, and poor learning enthusiasm (Ye, 2020). The above problems may be because teachers do not play a good guiding role, teachers’ personal ability is insufficient, and teaching methods are single, only focusing on theory and ignoring practical teaching. The popularization of computers has brought new ideas to reform music teaching. He (2021) argued that multimedia technology could enrich college music teaching resources and teaching forms and improve classroom efficiency (He, 2021). Song (2021) found that the music quality of contemporary college students was generally low, and the basic music knowledge, skills, and mastery were undesirable. Applying computer and Internet technologies was a good entry point to improve music education (Song, 2021). Huang (2021) designed an interactive creation platform based on music Computer-aided Instruction (CAI). The simulation results showed that the music teaching platform had higher efficiency, creative level, interactivity, and stability (Huang, 2021). The experts and scholars mentioned above have studied music aesthetic teaching from different angles, using QS and simulation models, providing a rich theoretical basis and research methods. However, the deficiency lies in the relatively conservative and single research methods. This work combines the emotional teaching theory with the popular Deep Learning (DL) methods to study music aesthetic teaching and emotional visualization, which have certain timeliness and reliability.

At present, the computer system is widely used in music teaching. However, the traditional music CAI system only provides audio playback, recording, deductive evaluation, or music creation. It cannot perform emotional analysis and expression for the current music works. Therefore, it has not been fully played in aesthetic teaching. Based on this, this work first expounds on the related theories of emotional teaching. Then, a music emotion classification model based on Deep Neural Network (DNN) is proposed. Finally, based on the emotional teaching theory, a music emotion visual teaching system is designed on the DNN. The validity of the proposed music emotion visual teaching system is verified by design experiments. Several innovations can be noted in this work. Based on the existing emotional teaching, it carries out practical research, combines the mature emotional teaching mode with the music aesthetic teaching, and optimizes their combination. The main contribution is to provide important theoretical and technical support for upgrading the follow-up teaching system and improving the quality of music aesthetic teaching.

## Analysis of basic theory and establishment of model

### Emotional teaching theories

#### Emotional teaching psychology

Teaching is a special activity with teachers as the guide and students as the main body, and its objective is to impart knowledge through strong emotions (Kos, 2018). The essence of emotional teaching is that teachers integrate emotions into teaching activities at students’ cognitive levels, realizing the teaching objective and improving the teaching effect (Chen and Guo, 2020). However, some educators pursue “test scores” too much, and ignore emotional teaching, which makes students gradually forget the essence and happiness of learning and their academic achievements become fewer and fewer. On the contrary, if teachers can fully tap the emotional source of students in the classroom with correct guidance, students’ desire for knowledge will be aroused, and their understanding ability and qualities will be improved by connecting their emotional experience in the classroom with the actual emotional life (Hemmeter et al., 2018).

**Figure 1:**
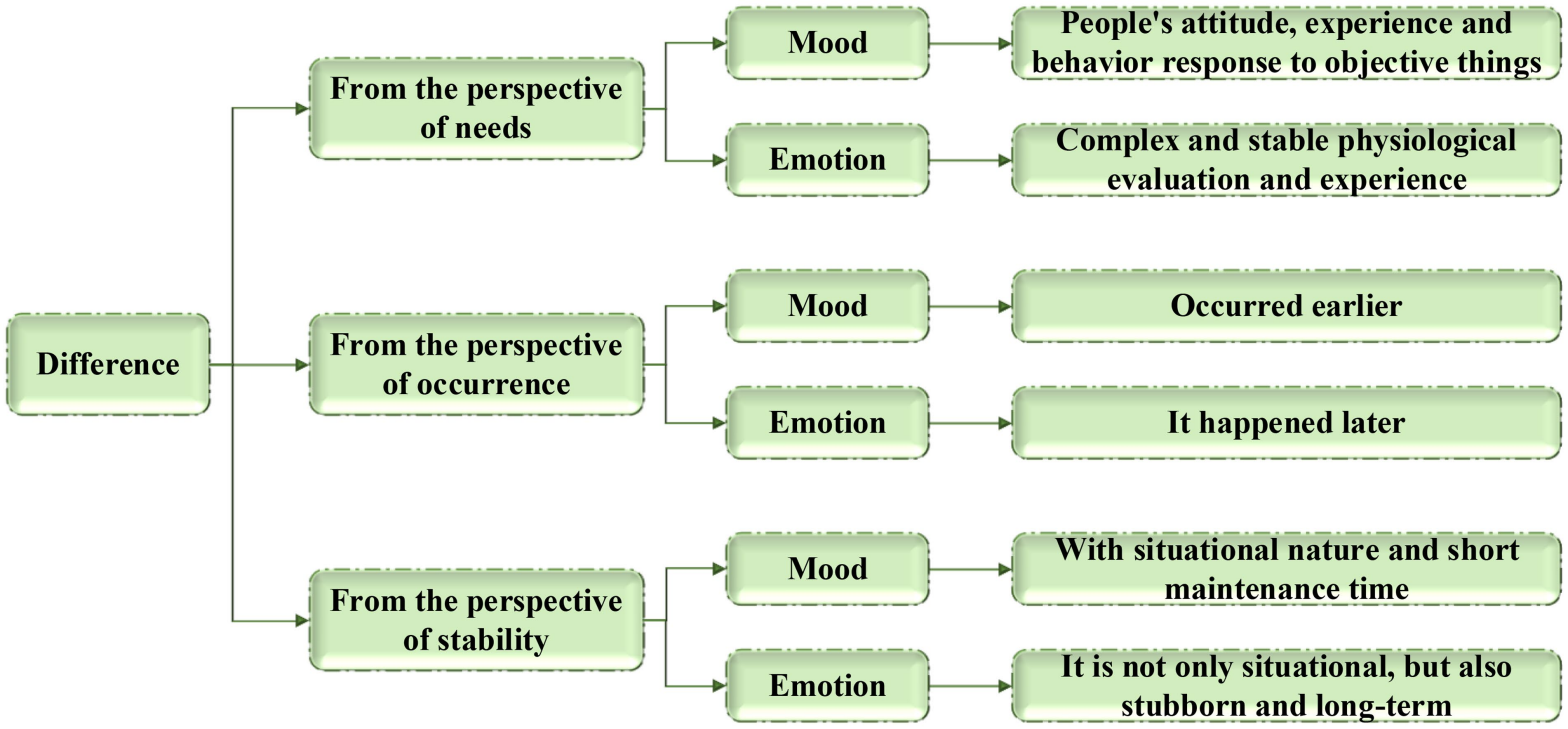
Difference between “moods” and “emotions.”

Emotional teaching psychology takes emotion teaching as the main body and it is based on teaching psychology. It aims to reveal some laws from some phenomena and in emotional psychology and update emotional teaching methods according to relevant theories of teaching psychology (Shen et al., 2017). Emotional teaching psychology mainly studies the three human psychological mechanisms of “moods,” “emotions” and “expressions.” Among them, “expression” is the most intuitive form to reflect human psychological emotions, and it is also the first step for teachers to understand students’ psychological states in the process of conveying knowledge. Students’ recognition of the information transmitted by teachers is reflected in their facial expressions, which will help teachers to change their teaching methods. “Moods” and “emotions” essentially refer to the same process and psychological phenomenon, but the former mainly reflects the psychological process, and the latter focuses on reflecting the psychological content (Chew et al., 2018). The difference between the two at different angles is shown in Figure 1.

Figure 1 shows that “mood” is often a short psychological process under the current situation, such as “joy” when you are happy, and “anger” when you are challenged, and it belongs to the attitude of people’s basic needs and desires. “Emotion” appears when you are engaged in social exchange for a period, and it is generalized as “love,” “happiness” and “beauty.” It belongs to the experience of social needs and desires. Therefore, emotion cannot be cultivated overnight. In the teaching process, teachers need to accurately know about the mood of students, mobilize students’ positive moods by establishing appropriate situations and taking correct guidance methods, and have an advanced emotional experience.

#### Music aesthetic psychology

The core of music teaching is to cultivate students’ positive and healthy aesthetic sentiment, aesthetic imagination, and aesthetic criticism, and help them cultivate their aesthetic values (Holochwost et al., 2017). Therefore, music teachers should introduce excellent music works with cultural connotations and rich emotions to students, analyze them carefully and convey them to students in a vivid way, so that students can fully understand the cultural connotation and emotions brought by music, and form “music aesthetics” in the teaching process. Music works symbolize beauty, and “music aesthetics” refers to people’s experience and perception of the beauty contained in music works and their cognition of different music cultural connotations (Koutsomichalis, 2018).

**Figure 2:**
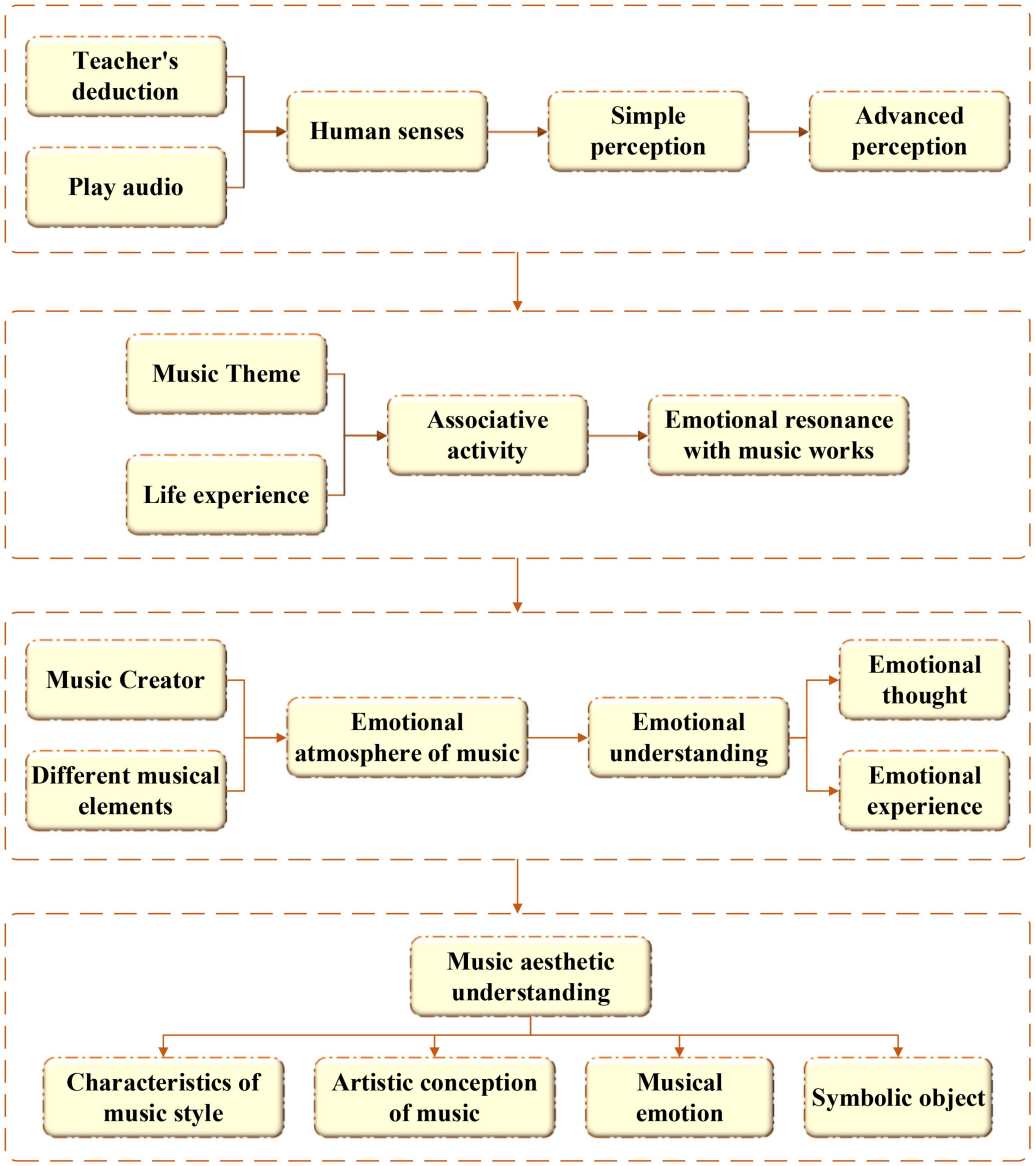
Relations of four psychological elements of aesthetic experience activities.

Music aesthetic psychology mainly explores students’ perception, understanding, and expression of music by fully understanding their psychological states when appreciating music. Here, the internal mechanism and characteristics of music aesthetic education are analyzed and studied, and a reliable and systematic method for aesthetic education is proposed, achieving the goal of reshaping students’ personalities, edifying the spiritual world, and cultivating talents with sound personalities for the country (Rozin and Rozin, 2018). The cultivation of music aesthetics should focus on both theoretical education and practical teaching in aesthetic experience activities. The four most basic psychological elements of aesthetic experience activities are “perception,” “association,” “emotion” and “understanding” and they are closely connected and complement each other to maximize the advantages of aesthetic experience activities (Hanrahan, 2018). The relationship between the four psychological elements of aesthetic experience activities is shown in Figure 2.

Figure 2 shows that the process of music aesthetic experience can be divided into four steps: music perception, emotional association, emotional resonance, and aesthetic understanding according to the development of their psychology. In the process, teachers often enable students to perceive the beauty of music works. Then, a series of association activities are carried out according to the music theme and their own real-life experience. Next, the students gradually resonate with the creator’s emotion to perceive the atmosphere that the creator hopes to convey through different musical elements. The styles, artistic conception, and emotions of music works are fully understood and grasped.

### Music emotion classification model based on DNN

**Figure 3:**
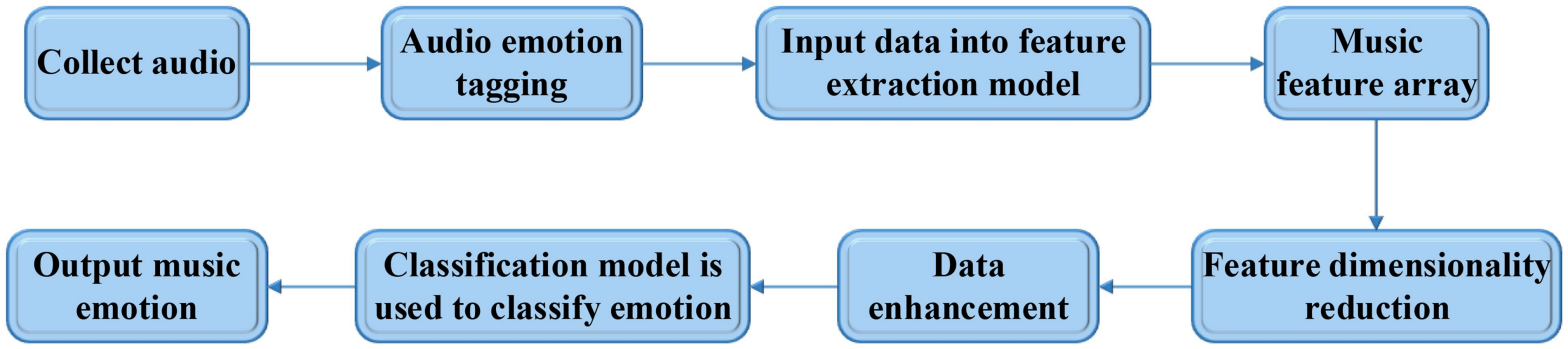
Flow chart of music emotion classification.

The key to music teaching is to promote students to establish their correct aesthetic values. In this process, teachers need to combine their own life experience and knowledge reserve to interpret the culture and emotion contained in a music work as deeply as possible, convey it to students, and deepen the students’ understanding through a series of aesthetic experience activities. This shows that music aesthetic teaching has high requirements for teachers’ comprehensive ability. However, different teachers have different understandings of the emotions in music works, and the emotions that they convey to the students in the music works have strong subjectivity, leading to the low accuracy of real emotions in music works. In view of this, music features are extracted and the emotions are classified based on deep learning, which relieves the teaching work by providing objective and reliable information, and the teaching effect is improved. The specific research process of music emotion classification based on DNN is shown in Figure 3.

#### Music feature extraction model based on the visual geometry group (VGG) convolution network

**Figure 4:**
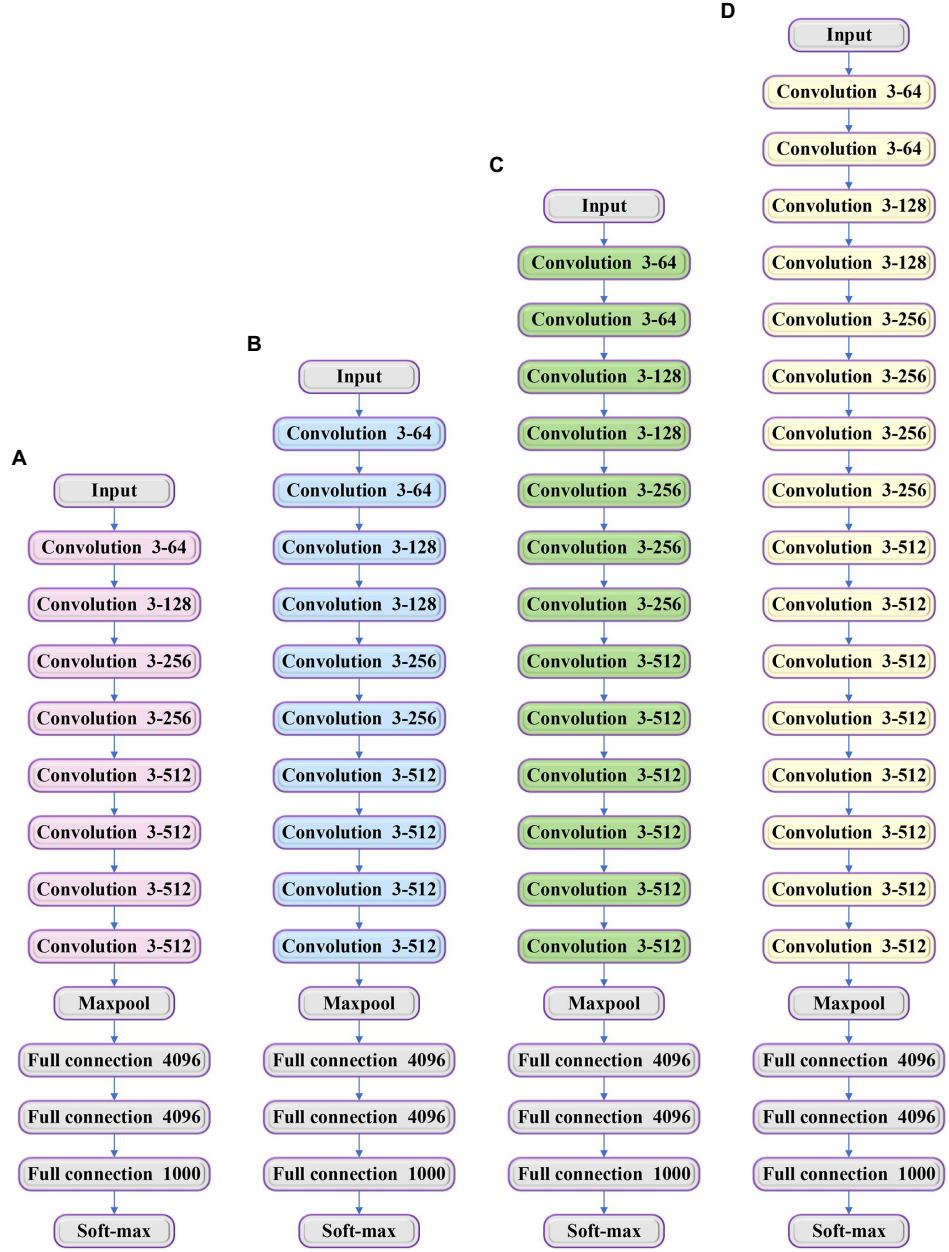
Structure of different types of visual geometry group (VGG; **A**) VGG-11; **(B)** VGG-13; **(C)** VGG-16; and **(D)** VGG-19.

Visual Geometry Group is used to extract the features of audios, and it can explore the influence of different convolutional neural networks (CNNs) on classification and recognition accuracy. VGG is divided into four types, namely VGG-11, VGG-13, VGG-16, and VGG-19, according to different network structures (Tammina, 2019). The network structure is shown in Figure 4.

**Figure 5:**
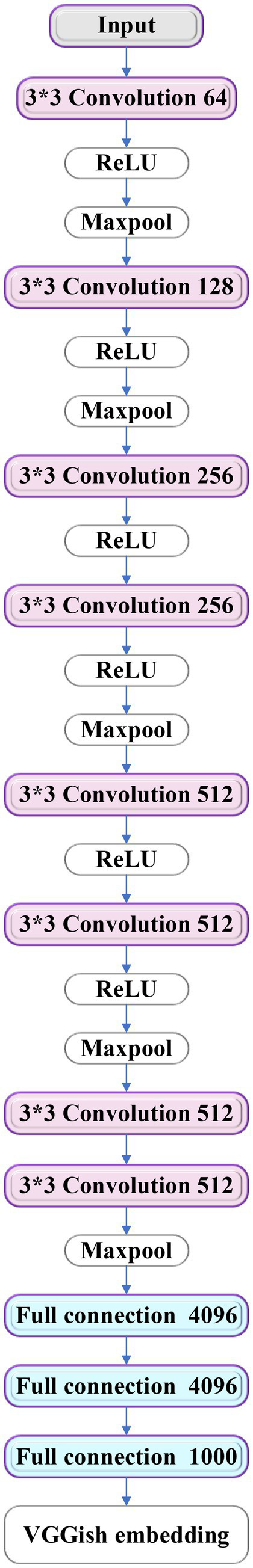
Structure of VGGish networks.

Figure 4 shows that the difference between the four types of VGG is that they have a different number of convolution layers, including 8 layers, 10 layers, 13 layers, and 16 layers. All the layers have three fully connected layers. The location and number of pooling layers need to be determined according to the actual needs. Within a reasonable range, the more layers the network has, the better the feature extraction effect is. The number of the networks of the traditional CNN cannot be added to more than ten layers, which can be overcome by VGG. At present, VGG networks are widely used in audio feature extraction. It is a derivative network of VGG (Ma et al., 2021), and its structure is shown in Figure 5.

**Figure 6:**
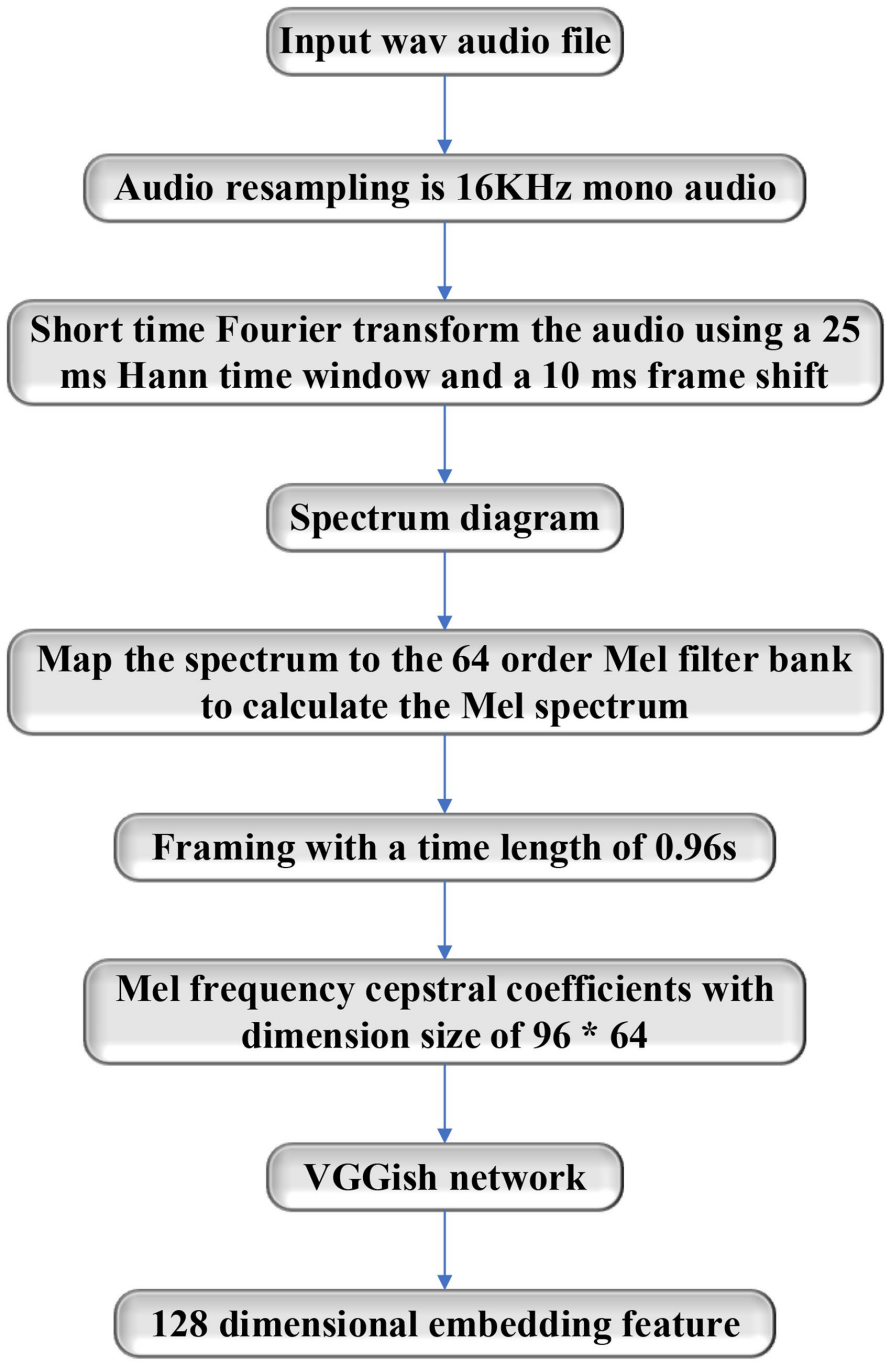
Flow chart of audio feature extraction.

The structure of VGGish networks is the same as that of VGG-11. It has eight convolution layers, five pooling layers, and three connection layers under different convolution layers. The convolution layer performs the task by using a 3*3 convolution core and Rectified Linear Unit (ReLU) activation function. The principle of extracting audio features using VGGish networks is that it can transform audio input features into 128-dimensional feature vectors with semantics, and then these feature vectors can be used as the input of the classification model. The audio feature extraction process is shown in Figure 6.

It needs to extract the Mel Frequency Cepstral Frequencies (MFCC) of audios before the feature is extracted by using VGGish networks (Fahad et al., 2021). The process is as follows: the audio is resampled; the Short-Time Fourier Transform (STFT) is conducted; Mel frequency is calculated. The calculation equation is as follows:


(1) $$logMel=\log\left(Melspectrum+0.01\right)\;$$

After the stable Mel frequency is obtained, the audio is divided into frames, the MFCC feature data with a dimension size of 96*64 are obtained and input into the VGGish network for feature extraction, and 128 dimensional feature vectors are achieved.

#### Data dimension reduction

The principle of Principal Components Analysis (PCA): The data in the original high-dimensional space contain a lot of redundant information and noise, which will have a great impact on the extraction accuracy of the model. Therefore, Principal Components Analysis (PCA) is employed to reduce the dimension of the data. PCA can extract the most representative feature vector in the feature set (Bhattacharya et al., 2020). The working principle of PCA is as follows:

If *A* = (*A*_1_, *A*_2_, *A*_3_, …, *A_N_*)*^T^* is a set of relevant variables affecting the research object, the variables can be transformed into the irrelevant variables *B* = (*B*_1_, *B*_2_, *B*_3_, …, *B_N_*)*^T^* through PCA. This process needs to meet the following two conditions:

1. Variance can reflect the amount of information transmitted by variables. Therefore, it requires that the variance of variables A and B should be equal before and after transformation to avoid the information loss in the process of a linear transformation, as shown in Equation (2).
(2) $$\overset{n}{\underset{i=0}{\sum }}variance\;\left(A_{i}\right)=\overset{n}{\underset{i=0}{\sum }}variance\;\left(B_{i}\right)$$
2. For converted *B*, it is necessary to make *B*_1_, *B*_2_, *B*_3_,…, *B_n_* linearly uncorrelated. *B*_1_, *B*_2_, *B*_3_,…, *B_N_* are all linear combinations of *A*_1_, *A*_2_, *A*_3_,…, *A_n_*. Variable *B*_1_ has the largest variance compared with other variables in *B* and becomes the first principal component. The variance of *B*_2_ ranks second, and it is the second principal component. In the final selection, the first m variables with high variance contribution are selected as long as they can show most of the information according to the order of principal components, and it does not need to use all *n* variables. The steps of PCA are as follows:

Before PCA, the obtained original data need to be standardized. At present, the most commonly used standardization method is *Z*-score (Simon et al., 2019), and its equation is as follows:


(3) $$Z=\frac{X-\bar{X}}{S}$$

In Equation (3), *Z* is the standardized original data, *X* is the corresponding indicator in the original data, and $\bar{X}$ is the average value of the indicator in the sample population.

*S* is the standard deviation of the indicator data.

**Figure 7:**
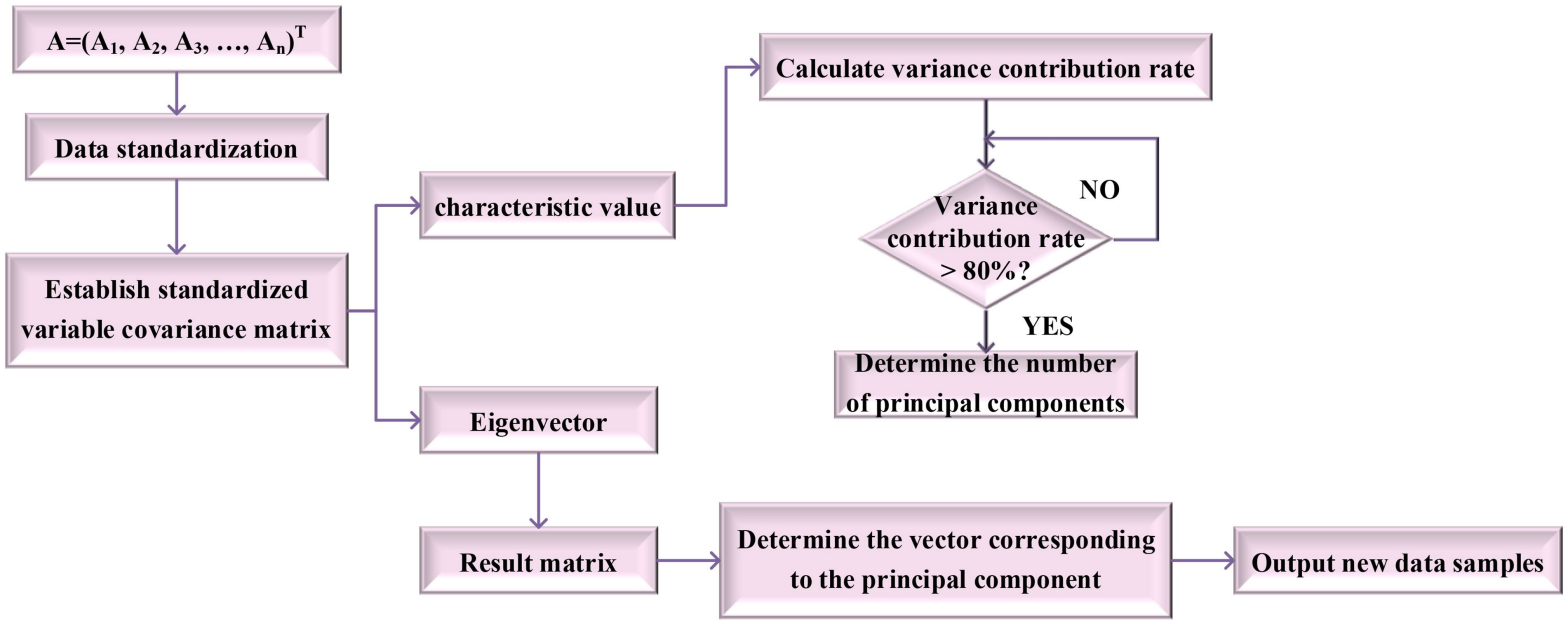
Main steps of principal components analysis (PCA).

All the data obtained can be dimensionless to avoid the impact of different dimensions. After the original data are processed, PCA is conducted. The details are as follows:

1. The processed data are arranged into matrix *X*
(4) $$X=\left[\begin{array}{ccc} x_{11} & \cdots & x_{1n}\\ \vdots & \ddots & \vdots \\ x_{k1} & \cdots & x_{kn} \end{array}\right]=\left[\begin{array}{c} X_{1}\\ \vdots \\ X_{k} \end{array}\right]$$
  In Equation (4), $x_{kn}$ is the new indicator after the original data are processed, *n* is the number of indicators contained in each data, *k* is the total number of data.
  $X_{k}$ is a vector consisting of each row of data in a matrix.
2. Calculate the covariance matrix Y of matrix X:
(5) $$Y=\left[\begin{array}{ccc} y_{11} & \cdots & y_{1k}\\ \vdots & \ddots & \vdots \\ y_{k1} & \cdots & y_{kk} \end{array}\right]$$
  Y is a k-order real symmetric matrix with k real eigenvalues, and the eigenvectors corresponding to different eigenvalues intersect in pairs.
3. Calculate the eigenvalue of matrix Y and its corresponding unit eigenvector:
  The first p eigenvalues are sorted in descending order as $\lambda_{1},\lambda_{2},\ldots ,\lambda_{p}$. The eigenvector corresponding to $\lambda_{p}$ is $a_{p}$, the coefficient set of the obtained principal component index *B_p_* corresponding to the original index vector *A_p_*. Suppose:
(6) $$a_{p}={\left\{b_{1},b_{2},\cdots ,b_{p}\right\}}^{T}$$
  Then:
(7) $$Y_{p}=a_{p}B_{p}$$
  The contribution rate of the new comprehensive indicator *B_i_* to overall variance *g_j_* is:
(8) $$g_{j}=\frac{variance\left(B_{j}\right)}{\sum_{i=1}^{m}variance\left(B_{i}\right)}$$
  A high contribution rate indicates that the principal component contains more information.
4. Determine the number of principal component indicators.
  Usually, the cumulative variance contribution rate G(j) is used to judge the number of principal component indicators, and its calculation is shown in Equation (9).
(9) $$G\left(j\right)=\frac{\sum_{i=1}^{j}\lambda_{i}}{\sum_{k=1}^{m}\lambda_{k}}$$
  When G(j) > 80%, the value of j is the number of selected principal component indicators. Also, the unit eigenvector corresponding to the eigen-root (whose λ>1) can be selected to constitute a transformation matrix for principal component selection. The number of principal components is the number of eigen-roots that meet the conditions. The main steps of PCA are shown in Figure 7.

#### Music emotion classification model based on Bi-LSTM

The recurrent neural network (RNN) has the function of applying the information learned from previous data to the current task (Majumder et al., 2019). However, the traditional RNN cannot effectively use the relevant information far away from the current prediction point, and it has much dependence. long short-term memory (LSTM) is proposed to solve this problem. Its calculation is performed by accumulation rather than continuous multiplication, and the derivative is also in the form of accumulation, which can effectively avoid the gradient disappearance or explosion (Fischer and Krauss, 2018). LSTM is composed of four elements: cell states, forgetting gates, input gates, and output gates (Le et al., 2019). Its structure is shown in Figure 8.

1. The forgetting gate directly determines whether the information in the unit state is passed or filtered. Its input has two parts. The first is h_t-1_ of the last time, and the other is input *x_t_* at the current time. Output *f_t_* of the forgetting gate is a vector whose value of each dimension is in the range of 0 ~ 1, b_*f*_ is an offset, *σ* represents a sigmoid function, and *W_f_* is the weight.
(10) $$f_{t}=\sigma \left(W_{f}*\left[h_{t-1},x_{t}\right]+b_{f}\right)$$
2. The input gate determines whether the information at the current time will be added to the unit state. Its input, like the forgetting gate, is *h_t_*–1 of the previous time and the input *x_t_* at the current time. The equations are as follows:
(11) $$i_{t}=\sigma \left(W_{i}*\left[h_{t-1},x_{t}\right]+b_{i}\right)$$
(12) $${\tilde{C}}_{t}=\tanh\left(W_{C}*\left[h_{t-1},x_{t}\right]+b_{C}\right)$$
  **Figure 8:**
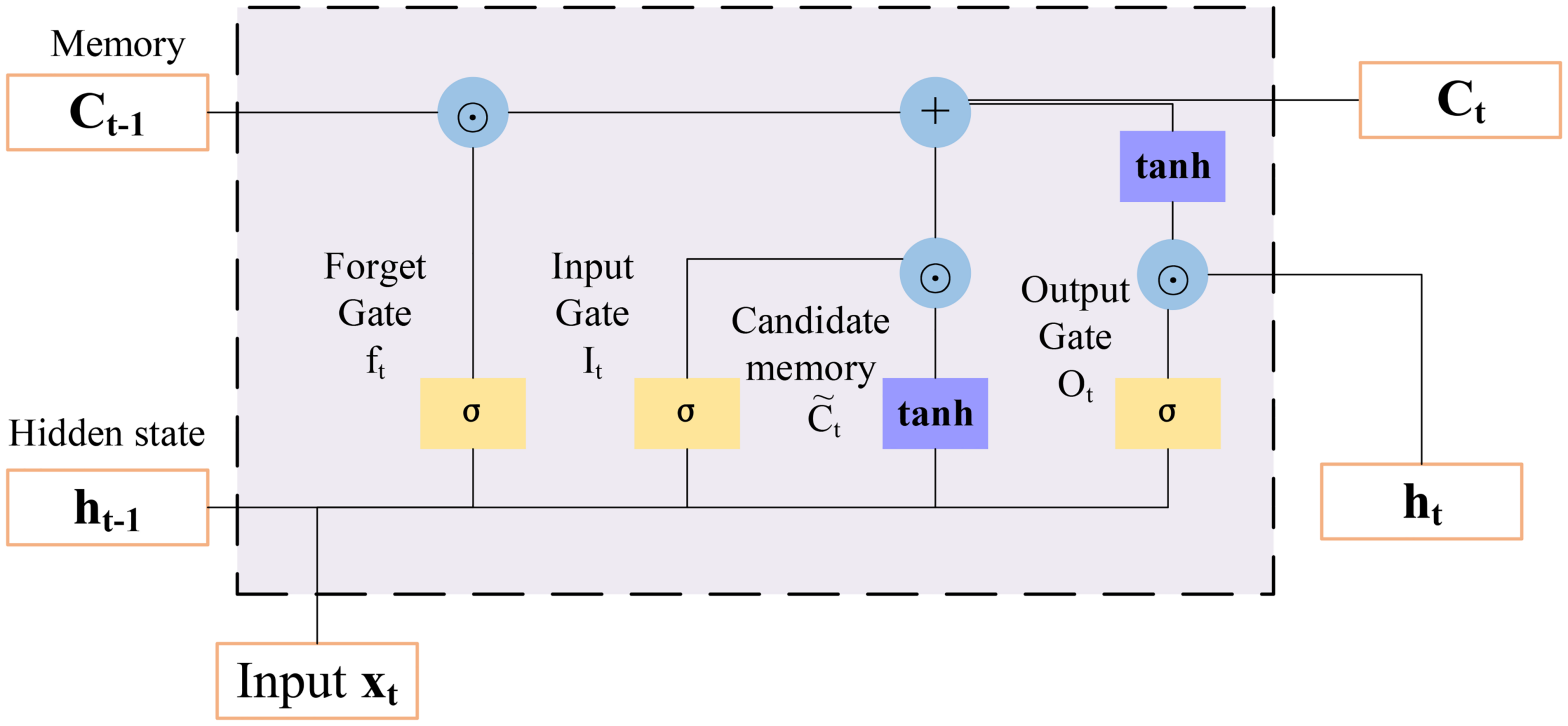
Structure of long short-term memory (LSTM).
  In Equations (11,12), *i_t_* is the input gate, and *C_t_* is the generated new cell state.
  (13) $$C_{t}=f_{t}*C_{t-1}+i_{t}*{\tilde{C}}_{t}$$
3. The output gate controls the output value at the current time, which depends on the input values of the previous time and the current time, as well as the new unit state at the current time:
(14) $$o_{t}=\sigma \left(W_{o}*\left[h_{t-1},x_{t}\right]+b_{o}\right)$$
(15) $$h_{t}=o_{t}*\tanh\left(C_{t}\right)$$

In Equations (14,15), *o_t_* stands for the output gate and *h_t_* represents the current output.

**Figure 9:**
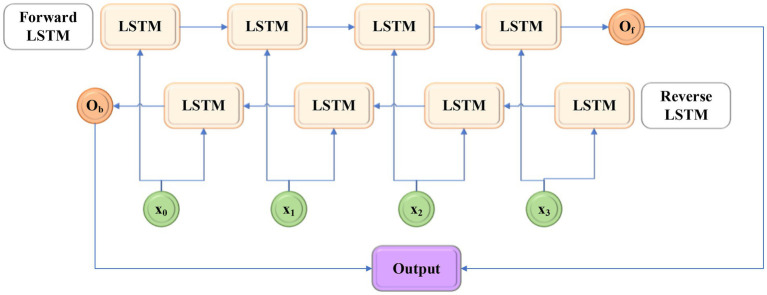
Structure of bi-directional long short-term memory (Bi LSTM).

In practical application, LSTM moves the input sequence from the beginning to the end, and it can also be called unidirectional LSTM. However, in the actual scene, the information of the whole sequence needs to be used, so bi-directional long short-term memory (Bi LSTM) comes into being. As the name suggests, Bi LSTM is composed of a forward-moving LSTM and a reverse LSTM (Li et al., 2019), and its structure is shown in Figure 9.

Figure 9 shows that *x_*0*_*–*x_*3*_* are the input, *O_f_* and *O_b_* are the outputs of the forward LSTM and reverse LSTM respectively, and the final output result is obtained by the joint action of the two. Here, the audio dataset with emotion labels is used to train the model, and the trained Bi LSTM is used to complete the classification of music emotion.

### Design of the visual system of music emotion teaching

**Figure 10:**
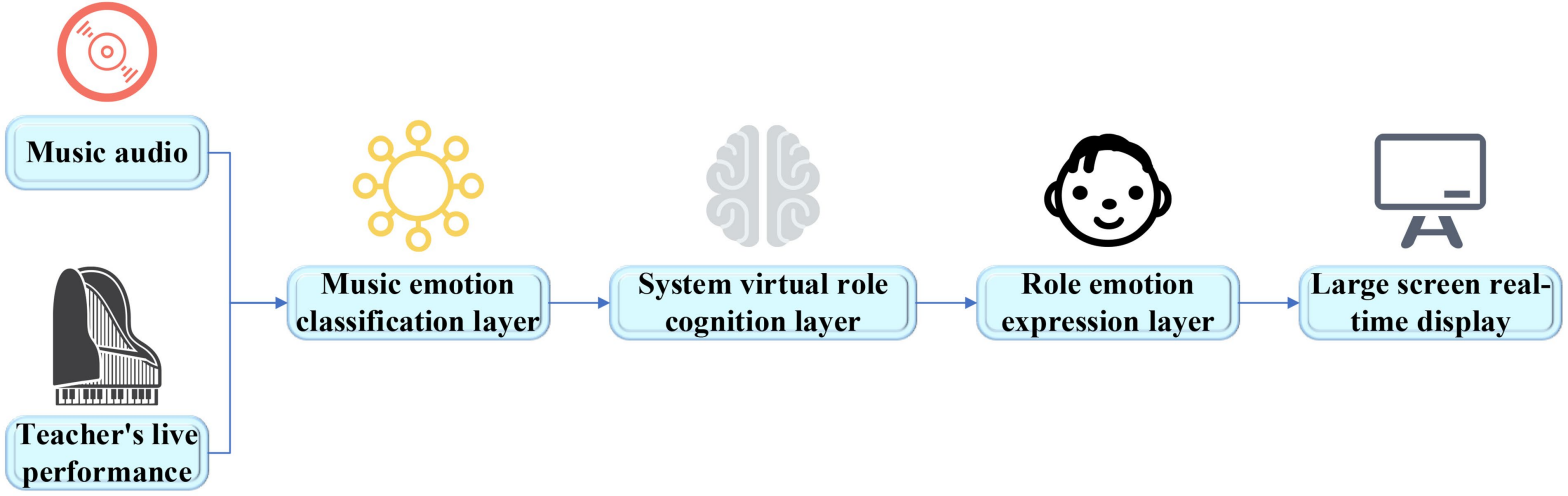
The architecture of the visual system of music emotion teaching.

Since the emotions in music aesthetic teaching are important, a music emotion classification model based on DNN is implemented. Based on the theory of emotion teaching and the model implemented, a visual system of music emotion teaching is designed to assist teachers to carry out high-quality music teaching. The basic architecture of the system is shown in Figure 10.

**Figure 11:**
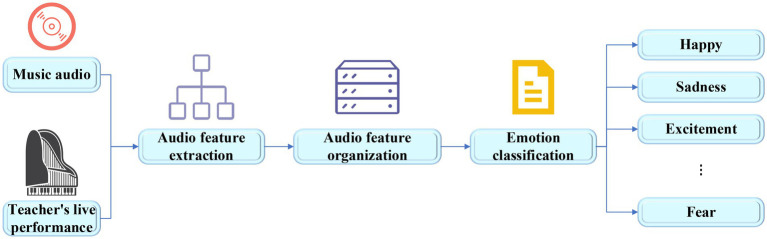
Flow chart of music emotion classification.

The system designed takes the audio of music works or the audio of teachers’ real-time musical instrument performance as the signal input, performs feature extraction and emotion classification in the music emotion classification layer, and transmits the types of music emotions to the cognitive layer and maps their virtual roles. The characters visually express the received emotions and display them in real time on the large screen of the system. Students can understand the emotion types to be expressed in the current work by observing the expression and behavior of the virtual characters. This visual teaching system can help teachers intuitively convey information to students, make it easy to understand the emotions in music works, which is helpful to the improvement of students’ aesthetic ability. The music emotion classification is shown in Figure 11.

The visualization of music emotion needs to be realized through the connection between emotions and virtual character behavior. In the system design, the relationship between virtual characters and emotion labels is established first. For example, when the emotional information of “happiness” is received, the virtual character shows a happy state in expression and behavior; If the message of “sadness” is received, it shows a sad state. The same label can establish a variety of expressions to enrich the images of the character. The behavior expression of virtual characters will be realized through computer animation (CA). CA can be divided into five types: key frame animation, deformation animation, process animation, joint animation, and physical animation. The pose transformation of the character is controlled by the corresponding dynamic equation and realized by the massless spring model, which is called physical animation (Ahmad et al., 2021).

### Design of the experiments

#### Dataset and data enhancement processing

**Figure 12:**
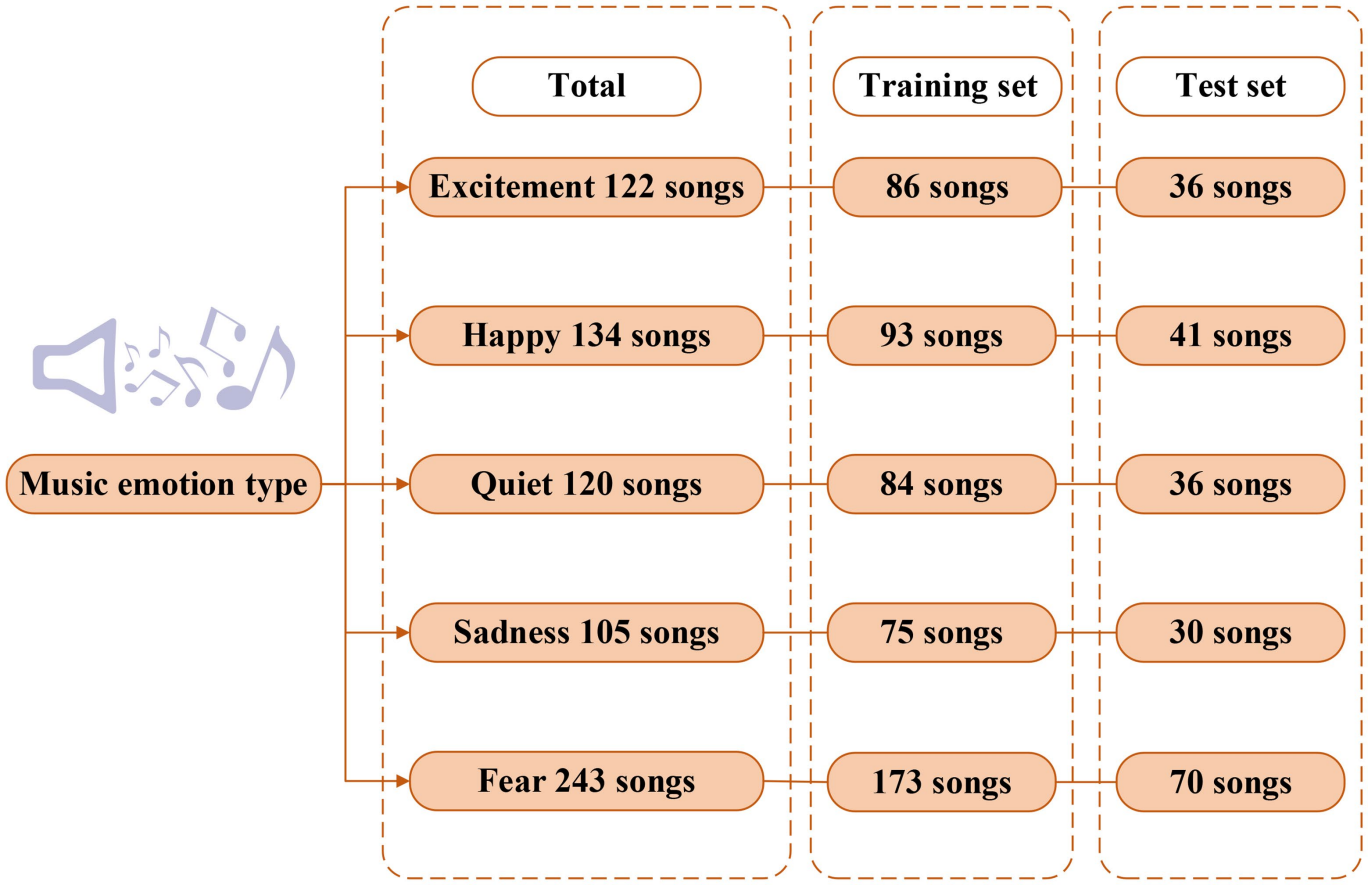
Music emotion distribution of the dataset.

About 1,200 audio recordings are downloaded from a music platform as experimental materials. The music on the platform has corresponding emotion labels. After the overlapped are excluded, five music emotion types of excitement, happiness, quiet, sadness, and fear are finally determined. Certain amounts of data are selected to construct the dataset. The dataset is divided into the training set and test set according to the ratio of 7:3. The specific distribution of each emotion type in the dataset is shown in Figure 12.

The audio data in the dataset are cut, translated, and noise-added. Data enhancement processing is to weaken the emotional features in music and explore whether the model can still accurately classify emotions under the condition of unclear features. The performance of support vector machine (SVM), CNN, and unidirectional LSTM is compared, and the classification accuracy is used as the indicator to explore the effectiveness of the Bi LSTM model. The precision of the models is shown as follows:


(16) $$Precision=\frac{TP}{TP+FP}$$

In Equation (16), TP is a true positive, and it means that the forecast is positive and the actual is positive; FP is false positive, which indicates that the forecast is positive and the actual is negative.

#### Optimization of model training p.arameters

The dataset constructed contains five types of emotions, so the number of input layer nodes of the LSTM network is five. The output is an emotion type, so the number of nodes in the output layer is one. The general calculation for the number of nodes in the hidden layer reads:


(17) $$M=\sqrt{NL}+\alpha$$

Here, M, number of hidden layer nodes. N, number of input layer nodes. L, number of output layer nodes. α, an integer ∈ [1, 10]. Here, the number of hidden layer nodes will be set as 5–11. The root mean square error (RMSE), mean absolute error (MAE), and Mean Square Error (MSE) in model training are taken as verification indicators to determine the optimal number of hidden layer nodes. The calculation of the three indicators is as follows:


(18) $$\mathrm{RMSE}=\sqrt{\frac{1}{n}\overset{n}{\underset{i=1}{\sum }}{\left({\hat{y}}_{i}-y_{i}\right)}^{2}}$$

(19) $$\mathrm{MAE}=\frac{1}{n}\overset{n}{\underset{i=1}{\sum }}\left|{\hat{y}}_{i}-y_{i}\right|$$

(20) $$\mathrm{MSE}=\frac{1}{n}\overset{n}{\underset{i=1}{\sum }}{\left({\hat{y}}_{i}-y_{i}\right)}^{2}$$

In Equations (18–20), *ny*_i_ is the predicted value, *y_i_* is the actual value, and *n* is the total number of samples.

A training cycle needs to completely process the dataset once, and the training times should be reasonable. The best training times are found through training. In the training process, if the learning rate is too small, the training will stop before finding the best parameters. If it is too large, it may cause oscillation or divergence. Therefore, it is necessary to find the best learning rate.

## Experimental results

### Optimization of the network parameters

**Figure 13:**
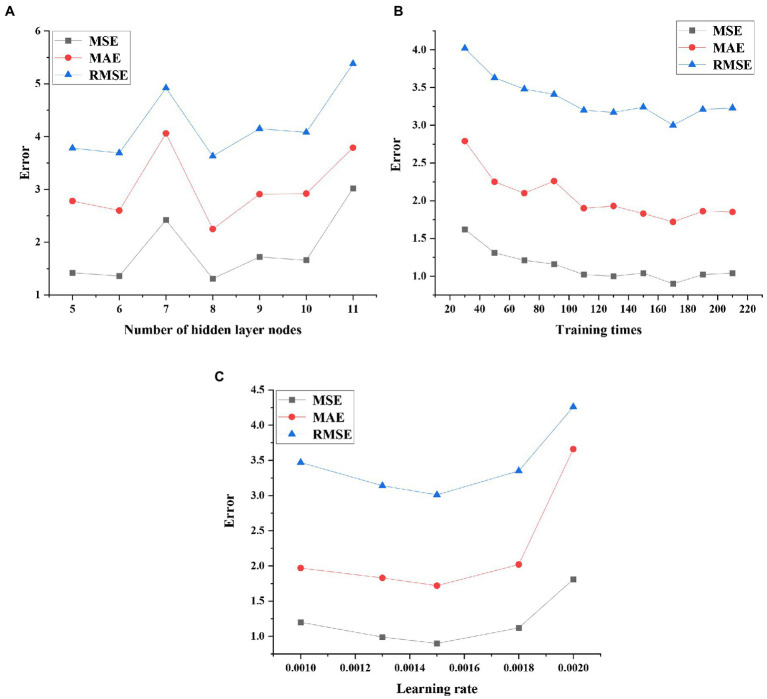
Performance test results of the model under different parameters **(A)** optimization results of hidden layer nodes; **(B)** optimization results of training times; and **(C)** optimization results of the learning rate.

The performance test results of the model under different hidden layer nodes, training times, and learning rate parameters are shown in Figure 13.

From the performance of the prediction model under different parameter settings, it is found that the performance of the model reaches the best when the number of hidden layer nodes is 8, the number of training times is 170 and the learning rate is 0.0015. Therefore, the optimal network parameter settings are shown in Table 1.

**Table 1:** Optimal parameters of long short-term memory (LSTM).

Parameters	Values
Number of the input layer nodes	5
Number of the output layer nodes	1
Number of the hidden layer nodes	8
Training times	170
Learning rate	0.0015

### Test results of the model

#### Test results on the original dataset

**Figure 14:**
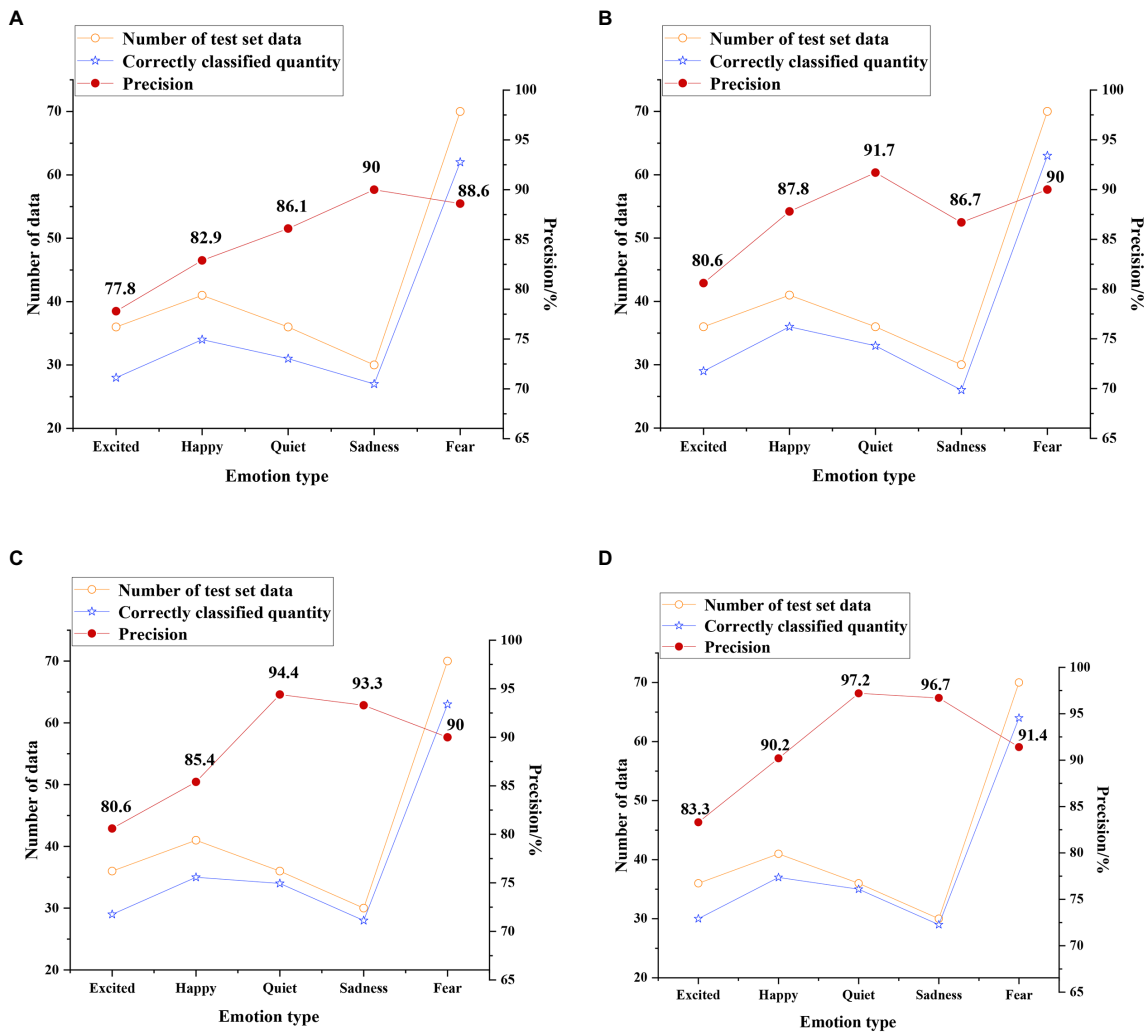
Test results on the original dataset **(A)** support vector machine (SVM) classification results; **(B)** convolutional neural network (CNN) classification results; **(C)** unidirectional LSTM classification results; and **(D)** Bi LSTM classification results.

The test results of the four classification models on the original dataset without enhancement processing are shown in Figure 14.

Figure 14 shows that the average precision of the four classification models of SVM, CNN, unidirectional LSTM, and Bi LSTM for the classification of five emotion types on the original dataset is 85.08, 87.36, 88.74, and 91.76%, respectively. This proves that the accuracy of the Bi LSTM model is more than 3.4% higher than other models, and its performance is better.

#### Test results on enhanced datasets

**Figure 15:**
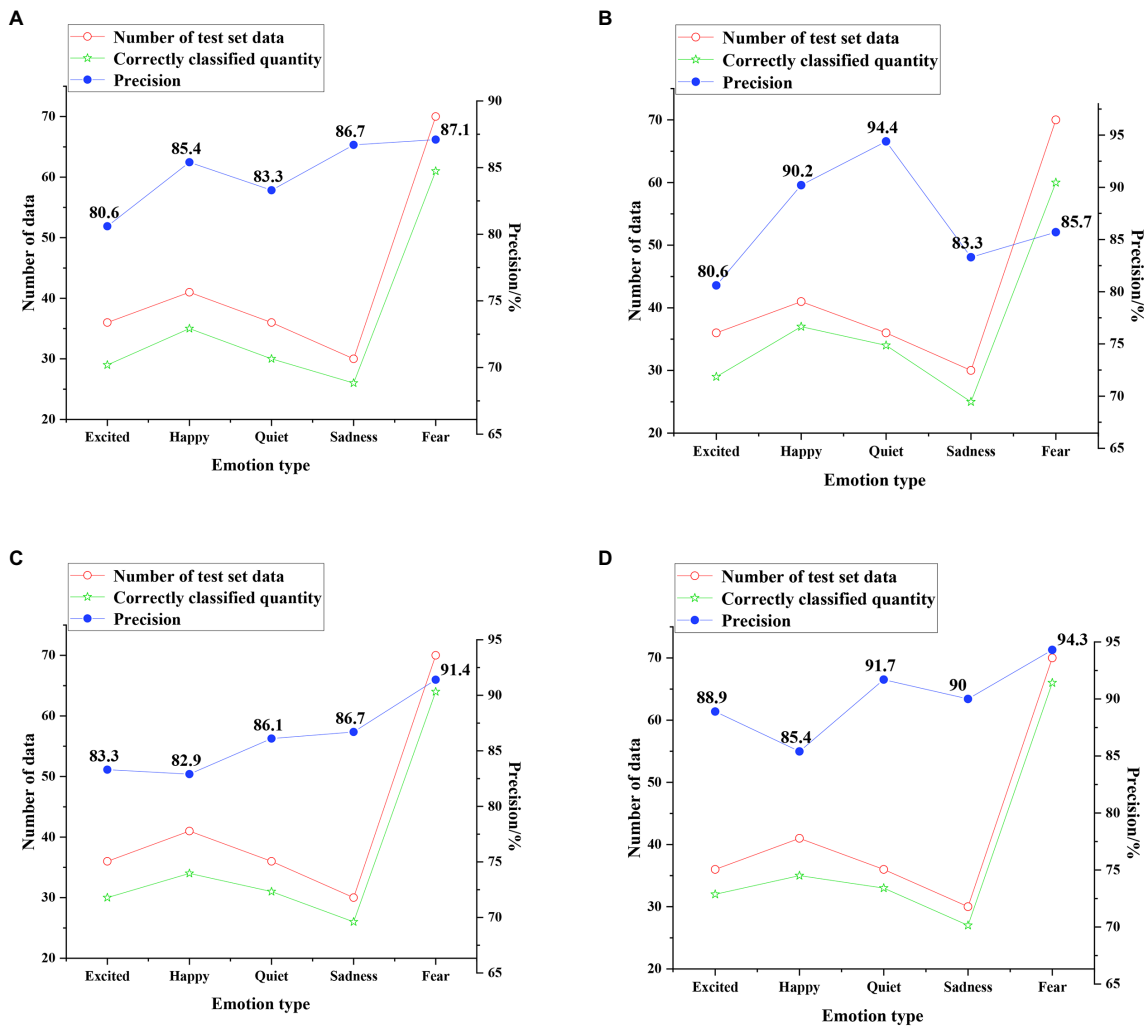
Test results on the enhanced dataset **(A)** SVM classification results; **(B)** CNN classification results; **(C)** unidirectional LSTM classification results; and **(D)** Bi LSTM classification results.

The test results of the four classification models on the enhanced dataset are shown in Figure 15.

The average precision of SVM, CNN, unidirectional LSTM, and Bi LSTM classification models for five emotion types on the enhanced dataset is 84.62, 86.84, 86.08, and 90.06%, respectively. This shows that the classification precision of each model decreases on the enhanced dataset, but the classification accuracy of the Bi LSTM model remains above 90%, which is more than 3.7% higher than that of other models. Therefore, the accuracy of the music emotion classification model based on DNN is more than 3.4% higher than other models, and the model has better performance. The visual system of music teaching for emotion classification can improve the effect of music aesthetic teaching.

## Conclusion

Music teachers have strong subjectivity and low accuracy in the interpretation of music works in the teaching process, and the traditional music teaching system cannot analyze and convey the emotion of works as required. Based on this, the relevant theories of emotional teaching are expounded, and a music emotion classification model based on DNN is implemented. Then, based on the emotional teaching theory and the model, a visual system of music emotion teaching is designed. The results show that the teaching system designed has five-layer, namely the audio input layer, emotion classification layer, virtual role perception layer, emotion expression layer, and output layer. The system can classify the emotions of the current input audio and map them to the virtual characters. Finally, they are displayed to the students through the display screen to realize the visualization of music emotions, so that the students can intuitively feel the emotions in the works. The accuracy of the music emotion classification model based on DNN implemented is more than 3.4% higher than other models, and the model has better performance. The music teaching system for emotion classification is appropriate for emotion classification. The shortcomings are that: only the music emotion classification technology is studied in detail, and the visualization system only provides a design scheme, which needs to be proved further. Future work should focus on the development and implementation of the system. The study provides important technical support for the upgrading of the teaching system and improving the quality of music aesthetic teaching.

## Data availability statement

The raw data supporting the conclusions of this article will be made available by the authors, without undue reservation.

## Ethics statement

The studies involving human participants were reviewed and approved by Shandong Normal University Ethics Committee. The patients/participants provided their written informed consent to participate in this study. Written informed consent was obtained from the individual(s) for the publication of any potentially identifiable images or data included in this article.

## Author contributions

The author confirms being the sole contributor of this work and has approved it for publication.

## Conflict of interest

The author declares that the research was conducted in the absence of any commercial or financial relationships that could be construed as a potential conflict of interest.

## Publisher’s note

All claims expressed in this article are solely those of the authors and do not necessarily represent those of their affiliated organizations, or those of the publisher, the editors and the reviewers. Any product that may be evaluated in this article, or claim that may be made by its manufacturer, is not guaranteed or endorsed by the publisher.
